# Bioaccessibility of Phenolic Compounds and Antioxidant Properties of Goat-Milk Powder Fortified with Grape-Pomace-Seed Extract after In Vitro Gastrointestinal Digestion

**DOI:** 10.3390/antiox11112164

**Published:** 2022-10-31

**Authors:** Danijel D. Milinčić, Nemanja S. Stanisavljević, Aleksandar Ž. Kostić, Uroš M. Gašić, Slađana P. Stanojević, Živoslav Lj. Tešić, Mirjana B. Pešić

**Affiliations:** 1 Institute of Food Technology and Biochemistry, Faculty of Agriculture, University of Belgrade, Nemanjina 6, 11080 Belgrade, Serbia; 2 Institute of Molecular Genetics and Genetic Engineering, University of Belgrade, P.O. Box 23, 11010 Belgrade, Serbia; 3 Department of Plant Physiology, Institute for Biological Research “Siniša Stanković”—National Institute of Republic of Serbia, University of Belgrade, Bulevar Despota Stefana 142, 11060 Belgrade, Serbia; 4 Faculty of Chemistry, University of Belgrade, Studentski Trg 12–16, 11000 Belgrade, Serbia

**Keywords:** pomace seed phenolics, goat milk, in vitro gastrointestinal digestion, phenolics-protein interaction, antioxidant properties, phenolics recovery

## Abstract

This study deals with the evaluation of the bioaccessibility and antioxidant properties of phenolic compounds from heat-treated skim goat-milk powder fortified with grape-pomace-seed extract, after in vitro gastrointestinal digestion. Ultra-high performance liquid chromatography coupled to diode array detection and mass spectrometry (UHPLC-DAD MS/MS) analysis confirmed the abundant presence of phenolic acids and flavan-3-ols in the grape-pomace-seed extract (SE) and heat-treated skim goat-milk/seed-extract powder (TME). After in vitro digestion of TME powder and recovery of total quantified phenolics, flavan-3-ols and phenolic acids were 18.11%, 24.54%, and 1.17%, respectively. Low recovery of grape-pomace-seed phenolics indicated strong milk protein–phenolic interactions. Electrophoretic analysis of a soluble fraction of digested heat-treated skim goat milk (TM) and TME samples showed the absence of bands originating from milk proteins, indicating their hydrolysis during in vitro gastrointestinal digestion. The digested TME sample had better antioxidant properties in comparison to the digested TM sample (except for the ferrous ion-chelating capacity, FCC), due to the presence of bioaccessible phenolics. Taking into account the contribution of the digestive cocktail, digested TME sample had lower values of total phenolic content (TPC), in vitro phosphomolybdenum reducing capacity (TAC) and ferric reducing power (FRP), compared to the undigested TME sample. These results could be attributed to low recovery of phenolic compounds. TME powder could be a good carrier of phenolics to the colon; thus, TME powder could be a promising ingredient in the formulation of functional food.

## 1. Introduction

During winemaking, a significant amount of grape seeds can be separated from pomace and utilized as a rich source of valuable phenolic compounds, primarily flavan-3-ols, phenolic acids, and procyanidins [1,2]. Previous studies have demonstrated that grape seed phenolic extracts exhibit a wide spectrum of beneficial effects due to their excellent antioxidant properties, reducing the risk of several chronic diseases such as cardiovascular diseases, inflammatory diseases, cancer, and diabetes [3,4,5,6]. For this reason, there is an increasing interest in the food sector in the production of functional food enriched with extracts obtained from winemaking by-products such as grape seeds or grape skin [7,8]. During the past decades, whole seed powder or extracts have been successfully incorporated into different cereal-based and milk-based products, which have been extensively studied and well reviewed [7,9,10,11,12]. Previous studies have recorded the low recovery of total phenolics, flavan-3-ols, or procyanidins, after in vitro gastrointestinal digestion of grape seed powder or extract in the absence of food matrices [13,14,15,16,17] or in the presence of a meat- and cereal-based food matrix as well [17]. Apart from this, only a few studies provide preliminary results for the antioxidant potential of in vitro digested cow milk-grape juice beverages [18,19,20]. However, the majority of studies conducted used cow’s milk, while the bioaccessibility of phenolic compounds in the presence of goat milk has been scarcely studied until recently [20,21]. In our previous research, goat-milk powders fortified with different concentrations of grape-pomace-seed extract have been developed, and their antioxidant properties have been examined [22]. However, the data considering the bioaccessibility of phenolics and antioxidant activity in the newly developed digested product are still lacking. To the best of our knowledge, the effect of goat milk or milk proteins on the bioaccessibility of phenolics from grape pomace seed has not been investigated so far.

Thus, this investigation aimed to evaluate the bioaccessibility of phenolic compounds and the antioxidant potential of goat-milk powder fortified with grape-pomace-seed extract, after in vitro gastrointestinal digestion.

## 2. Materials and Methods

### 2.1. Preparation of SE and TME Powder

Seeds separated from Prokupac grape pomace were ground and extracted with 80% methanol (80/20, water:methanol) [3]. The collected supernatant was evaporated to dryness, reconstituted in milliQ water (0.055 μS/cm), and used for the formulation of TME powder.

Heat-treated skim goat milk fortified with 0.6 mg TPC per mL milk was prepared according to the procedure described in our previous study [22]. This powder was selected for in vitro gastrointestinal digestion, due to having the best antioxidant properties among three tested milk/grape-pomace-seed extract powders analyzed in a previous study.

### 2.2. Simulated In Vitro Gastrointestinal Digestion

Standardized static in vitro digestion was conducted following the guidelines of the widely accepted methodology developed during the INFOGEST COST project [23]. Mixtures of samples and distilled water (5 g), containing 1 g of sample and 4 g water, entered the oral phase of digestion by the addition 3.5 mL of artificial salivary fluid (SSF) and 0.5 mL of α-amylase (1500 U/mL), 25 µL of 0.3 M CaCl_2_, and 975 µL of water. The mixture was homogenized thoroughly by shaking and incubated at 37 °C for 2 min. In the next phase of gastric digestion, 10 mL of the oral digest was added to 7.5 mL of artificial gastric fluid (SGF) and supplemented with 1.6 mL of pepsin (25,000 U/ mL), dissolved in SGF and 5 µL of 0.3 M CaCl_2_. The pH of the mixture was adjusted to 3.0 with HCl and distilled water was added up to 20 mL. Incubation was carried out at 37 °C for 2 h on a shaker at 300 rpm. After gastric phase was completed, an intestinal phase was started by adding 11 mL of artificial intestinal fluid (SIF), 5 mL pancreatin solution (800 U/mL) dissolved in SIF, 2.5 mL of bile salts (160 mM), and 40 µL of 0.3 M CaCl_2_. Sodium hydroxide solution was used to adjust the pH to 7.0 and distilled water was added to the mixture up to 40 mL.

Samples were then incubated at 37 °C for 2 h on a shaker at 300 rpm. After completing in vitro digestion, the supernatant containing the bioaccessible fraction was separated by pre-chilled (4 °C) centrifuge (5804R, Eppendorf, Hamburg, Germany) at 4500× *g* for 10 min and preserved at −80 °C for further analyses. The detailed composition of the artificial digestion fluids is provided in Table 1.

### 2.3. UHPLC-DAD MS/MS Analysis of Phenolic Compounds

The detection and quantification of phenolic compounds in samples before and after in vitro gastrointestinal digestion were conducted according to the method previously described by Pešić et al. [17] using a Dionex Ultimate 3000 UHPLC system equipped with a diode array detector (DAD) and TSQ Quantum Access Max triple-quadrupole mass spectrometer (ThermoFisher Scientific, Basel, Switzerland). Xcalibur software (version 2.2) (ThermoFisher Scientific, Inc., Waltham, MA, USA) was used for instrument control and the evaluation of phenolic compounds. Identification of phenolic compounds was performed by direct comparison with the commercially available standards purchased from Sigma-Aldrich (Steinheim, Germany). The quantification of phenolic compounds was performed by calculating peak areas and expressed as µg/L. LC/MS method validation parameters (retention time, regression equations, R^2^, and LOD and LOQ) are shown in Table S1 (Supplementary Materials).

### 2.4. Bioaccessibility

The bioaccessibility of detected phenolic compounds was calculated according to Pešić et al. [17]. Recovery of the total phenolics/class of phenolics and individual phenolics at the end of digestion were evaluated in relation to their amounts in SE:

(1) $$Recovery\;\left(\%\right)=\left(\frac{PC_{D}}{PC_{SE}}\right)\times 100,$$

Relative amount (%) of the total phenolics/class of phenolics and individual phenolic compounds bound to milk (*MBP*), were calculated with the following equation:

(2) $$MBP\left(\%\right)=\left(\frac{PC_{SE}-PC_{TME}}{PC_{SE}}\right)\times 100,$$

Relative amount (%) of the total phenolics/class of phenolics and individual phenolic compounds in the digested TME sample were evaluated in relation to their amounts in the reconstituted TME powder, according to the following equation:

(3) $$QP\left(\%\right)=\left(\frac{PC_{D}}{PC_{TME}}\right)\times 100,$$

where *PC_D_* is the content of the total phenolics/class of phenolics and individual phenolic compounds in digested samples; *PC_SE_* is the content of the total phenolics/class of phenolics and individual phenolic compounds in the initial SE; *PC_TME_* is the content of the total phenolics/class of phenolics and individual phenolic compounds in the reconstituted TME powder.

### 2.5. Electrophoretic Analysis

The protein profile of TM and TME samples before and after in vitro gastrointestinal digestion was analyzed by SDS-PAGE in reducing and non-reducing conditions, as previously described by Pesic et al. [24]. For both methods, the same separating gel (12.5% *w/v*; pH = 8.85), stacking gels (5% *w/v*; pH = 6.8), and tris-glycine running buffer were used.

Initial and digested TM and TME samples were mixed with the SDS-R-PAGE and SDS-NR-PAGE sample buffers (1:1 *v/v*), intensively vortexed, and used for electrophoretic analysis. Aliquots of 25 µL samples (25 µL and 100 µL for digestive cocktail control, DCC) were loaded into the wells. At the end of analysis, gels were stained with Coomassie blue dye. After destaining, the gels were scanned and analyzed using GelAnalyser 19.1 software (www.gelanalyser.com).

### 2.6. Total Phenolic Content and Antioxidant Properties

The total phenolic content and antioxidant properties (TAC; FRP; ABTS^•+^ scavenging activity, ABTS^•+^; and FCC) of the digestive cocktail control and the initial and digested samples were evaluated according to previously described methods [17,21,22]. Experiments for TPC and all antioxidant methods were conducted in triplicate (*n* = 3) and results were expressed as mg GAE/100 mL (TPC), mg ascorbic acid (AA)/mL (TAC, FRP, ABTS) and mg EDTA/mL (FCC).

### 2.7. Statistical Analysis

All results were conducted in triplicate and presented as means ± standard deviation. The *t*-test was used to evaluate the statistical significance between the means (*p < 0.05*). Graphs were arranged in GraphPad Prism 6 software (San Diego, CA, USA).

## 3. Results and Discussion

### 3.1. Phenolic Composition of Digested TME Powder

Phenolic profiles and recovery of individual phenolic compounds of TM and TME powders after in vitro gastrointestinal digestion are presented in Table 2. UV chromatograms at 254 and 280 nm of the SME sample are shown in Figure S1 (Supplementary Materials). The main classes of phenolic compounds detected in the SE were phenolic acids (27.63%) and flavan-3-ols (69.23%), making up 96.86% of the total quantified phenolic compounds. Among the quantified phenolic acids, the most abundant was gallic acid (3444.81 ± 117.4 µg/L), while the dominant and only found flavan-3-ols were catechin (8282.64 ± 246.89 µg/L) and catechin gallate (688.84 ± 36.32 µg/L). Other phenolic compounds were detected in significantly lower concentrations. Similar results were reported by other authors who analyzed phenolic compounds of Prokupac seeds [25,26,27]. It should be emphasized that the mix of phenolic standards contained other flavan-3-ols, such as gallocatechin, epigallocatechin, and epigallocatechin-gallate, but they were not detected in any of the analyzed samples either before or after in vitro digestion. Furthermore, phenolic compounds were not detected in the TM sample, the digested TM sample, or the digestive cocktail control.

Compared to the phenolic compounds detected in the SE (12,959.12 µg/L), the addition of the SE to heat-treated skim goat milk (TME) provoked a significant decrease (at *p <* 0.05) of the total phenolic compound content (from 12,959.12 µg/L to 2367.76 µg/L), reflecting a significant decrease in the individual phenolic compound content. Since defatted goat’s milk was used, it can be assumed that milk proteins bound 79.18% of phenolic acids, and 83.39% of flavan-3-ols, which makes in total 81.73% of all quantified phenolic compounds. The decreased content of the total quantified phenolic compounds was confirmed in several studies, after mixing pollen with goat’s milk (76.19%) [21], cinnamon extract with yogurt (64.8%) [28], fruit juice with cow’s milk (55%) [29], or a cinnamon-based drink with milk (45.8%) [30]. Phenolic compounds are known to have a high binding affinity for milk proteins [31,32], especially in the case of thermally treated milk proteins [33,34], due to the increased number of hydrophobic groups, available after the denaturation of whey proteins and the formation of WP/CN complexes [35]. However, the interactions of phenolic compounds with milk proteins are variable and highly dependent on the structure, molecular weight, and polarity of individual phenolic molecules [32,36,37], which was confirmed by the data obtained for MBP (Table 2).

Among the phenolic acids, only the content of caffeic acid in the digested TME sample did not significantly change in comparison to its content in the undigested TME sample. After in vitro gastrointestinal digestion, caffeic acid recovery (calculated in the relation to the content in the SE) was 57.17%. Different values of in vitro recovery of caffeic acid (calculated in the relation to the starting extract/s) can be found in the literature, for example, 75.2% in the fruit juice-milk model beverage [38], ~55–62% for different thermally treated coffee-skimmed cow’s milk model beverages [39], or ~25–100% for differently processed model coffee-whole milk beverages [40]. On the other hand, gallic and protocatehuic acids were not detected in the digested TME sample, which may be due to their reduced stability in the simulated gastrointestinal conditions or the formation of complex phenolic derivatives through polymerization, epimerization, and autoxidation reactions [38]. The final recovery of quantified phenolic acids depended only on the content of caffeic acid and amounts to 1.17%.

From eight quantified flavonoids in the SE, at the end of the in vitro TME digestion, only catechin, catechin-gallate, quercetin-3-glucoside, apigenin-7-glucoside, and aesculetin were detected. The absence or decreased recovery of some polyphenols at the end of in vitro TME digestion may be due to their reduced stability in the gastrointestinal conditions, degradation, or interactions with enzymes [30,41,42]. It has been previously demonstrated that milk proteins hydrolyze and lose their high affinity for binding polyphenols during in vitro gastrointestinal digestion, and partially release into the digestive fluid. On the other hand, a part of the polyphenols was probably retained in the insoluble fraction of the digested sample removed after in vitro gastrointestinal digestion. Moreover, Pineda-Vadillo et al. [43] showed that as much as 76.9% of the total phenolics remained in the insoluble fraction after the digestion of a milkshake enriched with grape pomace extract (Eminol^®^). Among the detected phenolic compounds, only the catechin content in the digested TME sample was significantly higher in comparison to the undigested TME sample. This result indicated that catechin was strongly bound to the milk proteins and released after the hydrolysis of milk proteins in simulated gastrointestinal conditions. The final recovery of catechin and catechin gallate was 26.19% and 4.66%, respectively. It was previously shown that milk proteins (especially caseins) readily bind and form complexes with flavan-3-ols from tea, thereby limiting their bioavailability, absorption, and potential biological function [44,45]. Different recoveries of catechin and epicatechin can be found in the literature, depending on the food matrix. For example, Rodríguez-Roque et al. [38] reported significantly lower recovery of (+)-catechin (14.8%) in the dialyzable fraction, after in vitro gastrointestinal digestion of a fruit juice-milk beverage, while He et al. [18] showed a significantly higher recovery of epi-catechin after in vitro gastrointestinal digestion of a mixture of grape juice with skimmed milk (~40%) or (~60%) in case of whole milk.

Quercetin-3-glucoside is the only detected flavonol at the end of in vitro gastrointestinal digestion, with a recovery of 20.38%, probably due to degradation and/or poor solubility in the simulated intestinal fluid. Isorhamnetin-3-*O*-glucoside and kaempferol were detected in the SE, but not detected in the initial and digested TME samples. Their absence in the digested sample is probably due to their strong interaction and retention in the insoluble fraction separated after digestion. Previously, Antunes-Ricardo et al. [46], showed that different isorhamnetin glucosides have a high recovery after in vitro gastrointestinal digestion. Although in small quantities, apigenin-7-glucoside was detected in the SE and a digested TME sample, so it can be assumed that apigenin-7-glucoside was bound with milk proteins and released after their hydrolysis by proteinases. Interestingly, the esculetin content was higher in the digested TME sample than in the SE, and its recovery was 133.42%. This can be explained by the potential cyclization of released hydroxycinnamic derivatives under the intestinal phase of digestion [47], which were not quantified by HPLC DAD MS/MS due to the lack of standards. The final recovery of quantified phenolic compounds of grape-pomace-seed extract in the presence of defatted thermally treated goat’s milk was 18.11%. Only a low recovery of phenolic compounds was reported by Pineda-Vadillo et al. [43], for grape pomace extract in the presence of a milkshake matrix. Since the low recovery of phenolic compounds has been observed after in vitro gastrointestinal digestion, it can be assumed that most phenolic compounds remain in the insoluble fraction. This makes them potentially available for release during digestion in the colon, where they can exhibit a preventive function against colorectal carcinoma as suggested by Pérez-Ortiz et al. [48]. Other authors reported a significantly higher recovery of total HPLC-quantified phenolic compounds of pollen in the presence of goat’s milk (30.71%) [21], fruit juice phenolics in the presence of skimmed milk (29.84%) [29], or phenolics from cinnamon-based beverages in the presence of cow’s milk (62.9%) [30]. Different bioavailability (recovery) of phenolic compounds in the presence of milk, recorded by different authors, can most probably be attributed to the variety of applied in vitro gastrointestinal digestion models and different types of milk being used as a protein matrix, as well as to the variable structural characteristics, stability, and polarity of phenolic molecules [49].

### 3.2. Electrophoretic Analysis of Milk Proteins after In Vitro Digestion

**Figure 1:**
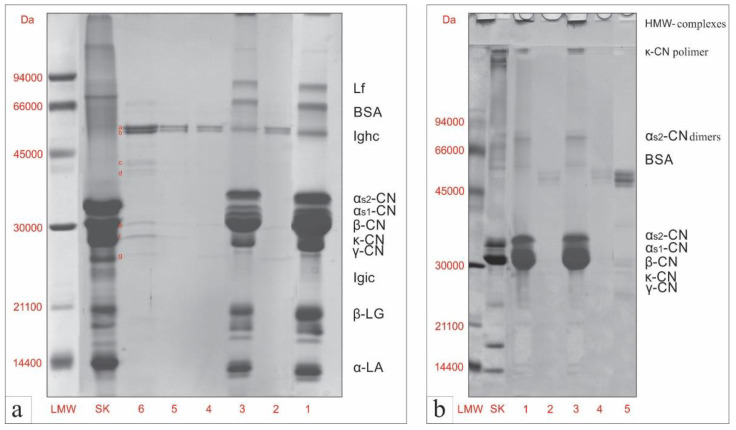
Electrophoretic patterns of TM and TME samples before and after in vitro gastrointestinal digestion, analyzed by SDS-R-PAGE (**a**) and SDS-NR-PAGE (**b**). Samples: TM—undigested heat-treated skim goat-milk powder (1); DTM—digested heat-treated skim goat-milk powder (2); TME—undigested heat-treated skim goat-milk/seed-extract powder (3); DTME—digested heat-treated skim goat-milk/seed-extract powder (4); digestive cocktail control, (25µL loaded into the well) (5); digestive cocktail control, (100 µL loaded into the well) (6); a,b,c,d,e,f,g—bands originating from enzymes. Abbreviations: molecular weight standard (LMW); bovine milk-protein standard (SK); lactoferrin (Lf); bovine serum albumin (BSA); immunoglobulin hard chain (Ighc); αs2-casein (αs2-CN); αs1-casein (αs1-CN); β-casein (β-CN), κ-casein (κ-CN); γ-casein (γ-CN); α-lactalbumin (α-LA); β -lactoglobulins (β -LG).

Protein profiles of TM and TME samples before and after in vitro gastrointestinal digestion (Figure 1, lines 1–4) were examined using SDS-PAGE under reducing (Figure 1a) and non-reducing (Figure 1b) conditions in the soluble fraction of samples. On the SDS-R-PAGE pattern, identical protein profiles can be observed for the TM and TME samples (Figure 1a, lines 1 and 3), with characteristic bands belonging to caseins and goat’s milk serum proteins. The only difference between these samples (TM and TME) is the intensity of the bands, as reported in our previous research [22]. Protein detection and characterization was performed using cow’s milk protein standards, molecular mass standards, and available data from the literature [22,24]. Apart from a few bands belonging to the enzymes from the digestive cocktail (Figure 1a, lines 5 and 6), at the end of the in vitro gastrointestinal digestion, there were no visible bands originating from the digested TM and TME samples (Figure 1a, lines 2 and 4). This indicated that the soluble fraction of digests had low-molecular-weight peptides and amino acids, released during in vitro gastrointestinal digestion, which could not be detected on a 12.5% (*w*/*v*) polyacrylamide gel. Similar results were reported by other authors, for in vitro digested skimmed cow’s milk [30,50], cinnamon-milk beverages [30], or goat milk without/with pollen [21], also using the standardized static COST INFOGEST digestion model. The intensive and diffuse bands in the digestive cocktail, with molecular masses of 56, 54, 43, 41, 28, 26, and 23 kDa (Figure 1a; line 6, lowercase letters next to bands), originated from pepsin, lipases, amylases, and proteinases of intestinal pancreatin [51].

The TM and TME samples also had characteristic SDS-NR-PAGE protein profiles (Figure 1b, lines 1 and 3), which are common for thermally treated goat milk [22,24]. At the end of in vitro gastrointestinal digestion, in both digested TM and TME samples (Figure 1b, line 2 and 4), only bands originating from the enzymes were visible, which is in agreement with the results of SDS-R-PAGE analysis. This means that the peptides released during in vitro gastrointestinal digestion did not subsequently form complexes in the soluble fraction of digests, which would potentially cause the appearance of some uncharacteristic bands or HMW complexes on the SDS-NR-PAGE patterns.

### 3.3. Total Phenolic Content and Antioxidant Properties of Digested Powders

**Figure 2:**
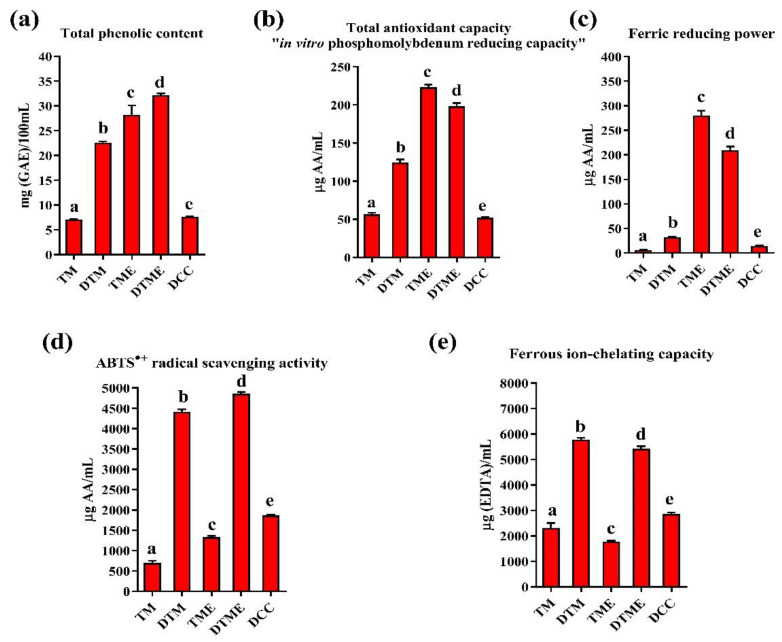
Total phenolic content (**a**) and antioxidant properties (**b**–**e**) of TM and TME samples before and after in vitro gastrointestinal digestion. Bars followed by the same lower letters are not significantly different according to *t*-test (*p* < 0.05), n = 3. Samples: TM—undigested heat-treated skim goat-milk powder; DTM—digested heat-treated skim goat-milk powder; TME—undigested heat-treated skim goat-milk/seed-extract powder; DTME—digested heat-treated skim goat-milk/seed-extract powder; DCC—digestive cocktail control.

The results for total phenolic content and antioxidant activity of the initial samples, in vitro digested samples and control of the digestive cocktail are shown in Figure 2a–e. The TPC of TM, DTM and DCC samples showed significant values, ranging from 7.64 to 22.61 mg (GAE)/100 mL, which is not in agreement with the results of the HPLC quantification of phenolic compounds. However, if the limitations of Folin–Ciocalteu’s reagent and its primary purpose in protein analysis are taken into account, the results obtained are most probably the consequence of the interference of lactose, enzymes, and peptides and amino acids that were formed during in vitro digestion [21,30]. The TME sample had a significantly higher TPC, in comparison to the TM sample, which was expected due to the presence of phenolic compounds from the SE. Similar observations were also reported by other studies for milk products fortified with phenolics from different plants [52,53]. At the end of in vitro gastrointestinal digestion, these compounds were liberated, contributing to the significantly higher TPC (32.18 ± 0.35 mg GAE/100 mL) of the digested TME sample, in comparison to the digested TM (22.61 ± 0.22 mg GAE/100 mL) sample. Similar results were reported by Helal et al. [30] for a digested cinnamon-milk drink, as well as Cilla et al. [54] for digested fruit juice-milk beverages. Furthermore, the digested TM and digested TME samples had a significantly higher TPC, in comparison to their undigested TM and TME samples. However, in this case, the influence of the digestive cocktail should not be ignored, because DCC, together with the released peptides, amino acids, and phenolic compounds, contributes to the higher TPC values of the digested samples [55]. If the contribution of these compounds to TPC values is taken into account, the digested TM sample had a higher, while digested TME lower, TPC, in comparison to the sum of TPC values for DCC and their undigested samples, TM and TME, respectively. The decreased TPC value of the digested TME sample in relation to the sum of TME and DCC samples could be due to the retention of phenolics in the insoluble fraction of digests as well as the reduced stability of released phenolic compounds in the simulated gastrointestinal conditions and their ability to easily polymerize and auto-oxidize [38]. These are in agreement with HPLC results, which also showed reduced bioavailability or complete absence of the majority of the phenolic compounds at the end of in vitro gastrointestinal digestion (Table 2). Similar to our results, Qie et al. [39] reported decreased TPC values for different models of skimmed milk-coffee beverages after in vitro gastrointestinal digestion.

The results for the in vitro phosphomolybdenum reducing capacity and Fe^3+^ reducing power of the analyzed samples are shown in Figure 2b,c, respectively. Both methods measure the metal ions’ reducing capacity and the obtained results were in good agreement. Differences in the recorded values for the analyzed samples between methods were due to the different methodologies. In the TAC method, bioactive compounds reduce Mo^6+^ ions in an acidic environment, while in the case of the FRP method, they reduce free Fe^3+^ ions. The TME sample showed significantly higher TAC and FRP values compared to the TM sample, due to the presence of phenolic compounds. Digestive cocktail control also showed reducing activity with TAC and FRP values of 52.26 ± 0.54 µg AA/mL and 14.61 ± 1.46 µg AA/mL, respectively. These values should be considered when interpreting the results obtained at the end of in vitro gastrointestinal digestion, because the digestive cocktail is an integral part of the digested TM and TME samples. The digested TM sample showed higher, while the digested TME sample had lower, TAC values, in comparison to the TAC values of their undigested samples or the sum of TAC values of DCC and TM or TME samples, respectively. However, the digested TME sample showed significantly higher TAC and FRP values, in comparison to the digested TM sample, due to the presence of bioavailable phenolic compounds. These results are in agreement with the TPC results. Nehir El et al. [51] reported significantly higher TAC values of digested goat kefir and milk in comparison to its undigested samples. On the other hand, Kostić et al. [21] showed a significantly lower TAC value for an in vitro digested goat milk-pollen powder, in comparison to its initial and control samples.

The ABTS^•+^ scavenging activity of the digested samples was significantly higher than ABTS^•+^ values for undigested samples or the sum of DCC and corresponding undigested samples (Figure 2d). Digested samples had better ABTS^•+^ scavenging activity, probably due to the release of low molecular weight peptides (not detected by SDS-R-PAGE) and amino acids during the enzymatic hydrolysis of goat-milk proteins [55]. During the in vitro gastrointestinal digestion, the peptides with high concentrations of tryptophan, cysteine, methionine, and histidine, which have a strong antioxidant capacity, were probably formed [19]. Furthermore, the digested TME sample had a significantly higher ABTS^•+^ scavenging activity, in comparison to the digested TM sample, indicating the additional contribution of the bioaccessible phenolic compounds to the radical quenching ability of the digested TME sample. The low ABTS value of the TME sample could be due to the presence of phenolic compound-WP/CN interactions that decreased the antioxidant activity of phenolics [22]. Several authors previously reported improved ABTS^•+^ activity after in vitro gastrointestinal digestion of grape juice-milk [18], cinnamon-milk [30], fruit juice-milk beverages [54], or yogurt enriched with cinnamon [28].

The TM and TME samples showed good chelating properties (Figure 2e), because they are protein matrices, which are generally known to be good chelating agents. Similar to our results, Shori [56] also reported good chelating capacity for milk products fortified with phenolic extracts of nutmeg and pepper. It is worth to mentioning that the digestive cocktail control showed better chelating properties, in relation to the TM and TME samples, which probably contributed to enzymes and salts from the digestive cocktail that can bind or react with ferrous ions. The digested TM (5783.27 ± 67.24 µg EDTA/mL) and digested TME (5424.64 ± 97.13 µg EDTA/mL) samples showed significantly higher FCC values, in comparison to their corresponding undigested samples or the sum of FCC values of DCC and their corresponding initial samples. This indicates that in vitro digestion of milk proteins probably liberated peptides with good chelating properties [55]. Some released phenolics during in vitro gastrointestinal digestion also can interact with enzymes and slow down milk-protein hydrolysis to amino acids, but chelation-capable peptides are formed [17,57].

## 4. Conclusions

This study investigated the bioaccessibility of phenolic compounds from goat-milk powder fortified with grape-pomace-seed extract before and after in vitro gastrointestinal digestion. The changes of the antioxidant potential were also monitored.

UHPLC-DAD MS/MS analysis of the SE and TME sample revealed the dominant presence of gallic acid and catechin in the samples. The content of total and all individual quantified phenolic compounds (except aesculetin) in TME sample was significantly decreased compared to their content in the SE (recovery ranged from 1.17 to 97.43), due to milk protein–phenolics interactions. After in vitro digestion, recovery of total phenolics and catechin was 18.11% and 26.19%, respectively. Gallic acid was not detected in digested TME sample. Low or absent recovery of total phenolics and individual phenolics could be due to their decreased stability and transformation in the intestinal fluid or retention in the insoluble fraction, which can be potentially available for the release in the colon phase. Electrophoretic analysis of the soluble fraction of digested TM and TME samples showed the absence of bands originating from milk proteins, indicating their hydrolysis during in vitro gastrointestinal digestion. All applied antioxidant assays (except FCC) exhibited good and higher values in digested TME than in digested TM samples, indicating good antioxidant activity of the bioaccessible phenolics. The digestive cocktail also showed antioxidant potential, which should be taken into account for the interpretation of results. Bearing in mind the contribution of DC to antioxidant properties of samples, it was shown that the digested TME sample containing the digestive cocktail had reduced TAC (for 34.56%) and FRP (for 30.45%) compared to the undigested sample. These could be due to the low recovery of phenolic compounds and their possible degradation and transformation during the applied digestion process. However, this can be considered beneficial, as phenolics retained in an insoluble fraction of digested TME sample could be liberated in the colon phase and exert their antioxidant properties. Thus, TME powder could be a potentially good carrier of phenolics to the colon, which should be examined in the future studies. In the conclusion, the enrichment of goat milk with grape-pomace-seed extract can be a promising ingredient in the formulation of functional food.

## Figures and Tables

**Table 1:** Composition of solutions applied in in vitro gastrointestinal digestion.

	SSF	SGF	SIF
	pH 7.0	pH 3.0	pH 7.0
Compound	Conc. in SSF mmol L^−1^	Conc. in SGF mmol L^−1^	Conc. in SSF mmol L^−1^
KCl	15.1	6.9	6.8
KH_2_PO_4_	3.7	0.9	0.8
NaHCO_3_	13.6	25	85
NaCl	-	47.2	38.4
MgCl_2_·(H_2_O)_6_	0.15	0.1	0.33
(NH_4_)_2_CO_3_	0.06	0.5	-
NaOH	-	-	8.4
HCl	1.1	15.6	-
* CaCl_2_·(H_2_O)_2_	0.75	0.075	0.3

Abbreviations: SSF—artificial salivary fluid, SGF—artificial gastric fluid, SIF—artificial intestinal fluid. SSF, SGF, and SIF were prepared as 1.25 × concentrates. * Concentrations of CaCl_2_·2H_2_O in final digestion mixture (added separately from the artificial fluids).

**Table 2:** The content and recovery of phenolics in SE, TM, and TME powders before and after in vitro gastrointestinal digestion.

Samples (µg/L)	SE	TM	DTM	TME	MBP %	DTME	QP %	Recovery %
**Phenolic acid**								
Gallic acid	3444.81 ± 117.4 ^a^	n.d.	n.d.	645.68 ± 20.12 ^b^	81.26	n.d.	0	0
Protocatehuic acid	62.50 ± 1.99 ^a^	n.d.	n.d.	57.02 ± 2.46 ^b^	8.77	n.d.	0	0
Caffeic acid	73.28 ± 4.30 ^a^	n.d.	n.d.	42.82 ± 2.38 ^b^	41.56	41.89 ± 3.55 ^b^	97.83	57.17
**Σ**	**3580.6 (27.63)**	**/**	**/**	**745.52 (31.49)**	**79.18**	**41.89 (1.78)**	**5.62**	**1.17**
**Flavan-3-ols**								
Catechin	8282.64 ± 246.89 ^a^	n.d.	n.d.	1444.85 ± 67.4 ^b^	82.56	2169.46 ± 81.50 ^c^	150.15	26.19
Catechin gallate	688.84 ± 36.32 ^a^	n.d.	n.d.	45.11 ± 0.113 ^b^	93.45	32.08 ± 1.43 ^c^	71.12	4.66
Galocatechin	n.d.	n.d.	n.d.	n.d.	-	n.d.	-	-
Epigalocatechin	n.d.	n.d.	n.d.	n.d.	-	n.d.	-	-
Epigalocatechin-gallate	n.d.	n.d.	n.d.	n.d.	-	n.d.	-	-
**Σ**	**8971.5 (69.23)**	**/**	**/**	**1490 (62.93)**	**83.39**	**2201.5 (93.50)**	**147.76**	**24.54**
**Other detected phenolics**								
Quercetin-3-glucoside	131.25 ± 6.66 ^a^	n.d.	n.d.	64.48 ± 3.94 ^b^	50.87	26.75 ± 1.88 ^c^	41.48	20.38
Isohramnetin-3-*O*-glucoside	49.82 ± 4.40	n.d.	n.d.	n.d.	100	n.d.	-	0
Kaempferol	123.93 ± 7.49	n.d.	n.d.	n.d.	100	n.d.	-	0
Apigenin-7-*O*-glucoside	16.08 ± 0.94 ^a^	n.d.	n.d.	n.d.	100	15.66 ± 2.02 ^a^	-	97.43
Naringenin	40.08 ± 4.55	n.d.	n.d.	n.d.	100	n.d.	-	0
Aeskuletin	45.89 ± 3.34 ^a^	n.d.	n.d.	67.79 ± 3.45 ^b^	-	61.23 ± 1.47 ^c^	90.32	133.42
**Σ**	**407.06 (3.14)**	**/**	**/**	**132.28 (5.59)**	**67.50**	**103.64 (4.42)**	**78.35**	**25.46**
**Total phenolic compounds**	**12,959.12**	**/**	**/**	**2367.76**	**81.73**	**2347.1**	**99.13**	**18.11**

Values are presented as means ± standard deviation. value in parenthesis represent relative amount of phenolic class in the sample. Different letters (a–c) in the same row denote a significant difference according to *t*-test (*p* < 0.05). n.d.—not detected. Abbreviations: SE—grape-pomace-seed extract; TM—undigested heat-treated skim goat-milk powder; DTM—digested heat-treated skim goat-milk powder; MBP—milk bound phenolic compounds; TME—undigested heat-treated skim goat-milk/seed-extract powder; QP—relative amount of phenolic compounds in digested TME sample (calculated in relation to the reconstituted TME powder); DTME—digested heat-treated skim goat-milk/seed-extract powder; Recovery—recovery of phenolic compounds after in vitro digestion of heat-treated skim goat-milk/seed-extract powder.

## Data Availability

No new data were created or analyzed in this study. Data sharing is not applicable to this article.

## Supplementary Materials

The following supporting information can be downloaded at: https://www.mdpi.com/article/10.3390/antiox11112164/s1, Figure S1: The UV chromatograms at 254 and 280 nm of TME sample (1—Gallic acid; 2—Protocatechuic acid; 3—Catechin; 4—Aesculetin; 5—Caffeic acid; 6—Quercetin 3-O-glucoside; 7—Catechin gallate); Table S1: The information on the characteristics of the LC/MS method (retention time, regression equations, R^2^ and LOD and LOQ).
