# Supplementary material for: Bioaccessibility of Phenolic Compounds and Antioxidant Properties of Goat-Milk Powder Fortified with Grape-Pomace-Seed Extract after In Vitro Gastrointestinal Digestion
Source: Antioxidants (Basel). 2022 Oct 31;11(11):2164. doi: 10.3390/antiox11112164 (PMC9686738; doi:10.3390/antiox11112164)
Supplement: Supplementary file 1 [file antioxidants-11-02164-s001.zip › antioxidants-1989665-supplementary.pdf]

Bioaccessibility of phenolic compounds and antioxidant properties of goat milk powder fortified with grape pomace seed extract after *in vitro* gastrointestinal digestion

Danijel D. Milinčić[1], Nemanja S. Stanisavljević[2], Aleksandar Ž. Kostić[1], Uroš M. Gašić[3], Slađana P. Stanojević[1], Živoslav Lj. Tešić[4], Mirjana B. Pešić[1]*

Table S1. The information on the characteristics of the LC/MS method (retention time, regression equations, R2 and LOD and LOQ)

Compound name	Retention time (min)	Linear regression (*Y*=*a*+*b***X*)	r^2	LOD (mg/L)	LOQ (mg/L)
**Gallic acid**	1.92	Y = - 39972.3 + 878796*X	0.9959	0.08	0.27
**Protocatechuic acid**	3.71	Y = - 117212 + 3417570*X	0.9942	0.10	0.33
**Caffeic acid**	5.53	Y = - 157197 + 10806700*X	0.9980	0.06	0.19
**Catechin**	4.99	Y = - 3539.10 + 3535423*X	0.9983	0.05	0.16
**Catechin gallate**	6.22	Y = - 92987.3 + 3017810*X	0.9961	0.08	0.26
**Gallocatechin**	3.72	Y = - 9632.86 + 187473*X	0.9986	0.05	0.16
**Epigallocatechin**	4.64	Y = - 4911.87 + 152360*X	0.9945	0.09	0.31
**Epigallocatechin gallate**	6.79	Y = 7027.22 + 363232*X	0.9929	0.10	0.32
**Quercetin 3-*O*-glucoside**	6.19	Y = - 53503.4 + 6308160*X	0.9926	0.11	0.36
**Isohramnetin 3-*O*-glucoside**	6.63	Y = - 16925.9 + 1161160*X	0.9992	0.04	0.12
**Kaempferol**	8.80	Y = - 43114.9 + 392203*X	0.9917	0.12	0.39
**Apigenin 7-*O*-glucoside**	6.91	Y = - 137958 + 9050410*X	0.9988	0.03	0.11
**Naringenin**	8.53	Y = - 225683 + 6063340*X	0.9921	0.11	0.38
**Aeskuletin**	5.30	Y = - 102582 + 6232950*X	0.9923	0.11	0.37

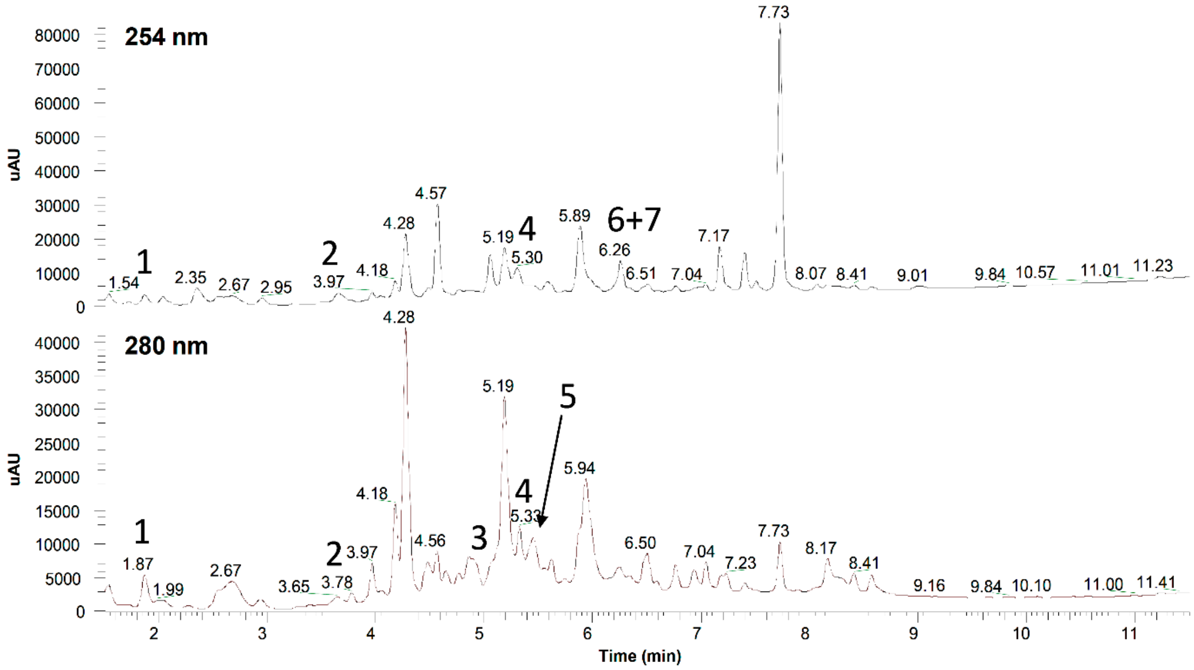

Figure S1. The UV chromatograms at 254 and 280 nm of TME sample (1 – Gallic acid; 2 – Protocatechuic acid; 3 – Catechin; 4 – Aesculetin; 5 – Caffeic acid; 6 – Quercetin 3-O-glucoside; 7 – Catechin gallate)
